# Women’s empowerment and male involvement in antenatal care: analyses of Demographic and Health Surveys (DHS) in selected African countries

**DOI:** 10.1186/1471-2393-14-297

**Published:** 2014-08-30

**Authors:** Larissa Jennings, Muzi Na, Megan Cherewick, Michelle Hindin, Britta Mullany, Saifuddin Ahmed

**Affiliations:** Department of International Health, Johns Hopkins Bloomberg School of Public Health, 615 N. Wolfe St., E5038, Baltimore, MD 21205 USA; Department of Biostatistics, Johns Hopkins Bloomberg School of Public Health, Baltimore, MD USA; Department of Population, Family, and Reproductive Health, Johns Hopkins Bloomberg School of Public Health, Baltimore, MD USA

**Keywords:** Sub-Saharan Africa, Women’s empowerment, Autonomy, Male involvement, Antenatal care, Pregnancy, Maternal health, Demographic health survey

## Abstract

**Background:**

Increasing women’s status and male involvement are important strategies in reducing preventable maternal morbidity and mortality. While efforts to both empower women and engage men in maternal health care-seeking can work synergistically, in practice they may result in opposing processes and outcomes. This study examines whether a woman’s empowerment status, in sum and across economic, socio-familial, and legal dimensions, is associated with male partner accompaniment to antenatal care (ANC).

**Methods:**

Women’s empowerment was measured based on the sum of nine empowerment items in the 2010–2011 Demographic and Health Surveys in eight sub-Saharan African countries: Burkina Faso (n = 2,490), Burundi (n = 1,042), Malawi (n = 1,353), Mozambique (n = 414), Rwanda (n = 1,211), Senegal (n = 505), Uganda (n = 428) and Zimbabwe (n = 459). In cross-sectional analyses, bivariate and multivariable logistic regressions models were used to examine the odds of male partner accompaniment to ANC between women with above-average versus below-average composite and dimensional empowerment scores.

**Results:**

In the majority of countries, male accompaniment to ANC was not uncommon. However, findings were mixed. Positive associations in women’s composite empowerment and male involvement were observed in Burkina Faso (OR = 1.27, 95% CI: 1.08, 1.50) and Uganda (OR = 1.53, 95% CI: 1.00-2.35), and in the economic empowerment dimension in Burkina Faso (OR = 1.24, 95% CI: 1.05-1.47). In Malawi, significant negative associations were observed in the odds of male accompaniment to ANC and women’s composite (OR = 0.77, 95% CI: 0.62-0.97) and economic empowerment scores (OR = 0.75, 95% CI: 0.59-0.94). No significant differences were observed in Burundi, Mozambique, Rwanda, Senegal, or Zimbabwe.

**Conclusion:**

Women’s empowerment can be positively or negatively associated with male antenatal accompaniment. Male involvement efforts may benefit from empowerment initiatives that promote women’s participation in social and economic spheres, provided that antenatal participation does not undermine women’s preferences or autonomy. The observation of mixed and null findings suggests that additional qualitative and longitudinal research may enhance understanding of women’s empowerment in sub-Saharan African settings.

## Background

Empowering women and increasing male involvement in maternal health care-seeking are both viewed as important strategies to reduce preventable maternal morbidity and mortality worldwide [1–3]. Each year, roughly a third of maternal deaths worldwide are directly related to inadequate care during pregnancy [1], and complications during pregnancy can result in acute and chronic maternal morbidity [4, 5]. Focused antenatal care, including identification and management of infections and obstetric complications, connects a woman and her household to formal health services and increases the likelihood of her giving birth with a skilled attendant [1, 6]. Antenatal care visits are an ideal time to advise mothers and families on essential pregnancy care to reduce stillbirths and neonatal deaths, and develop a birth preparedness plan [1]. In sub-Saharan Africa, approximately 69% of pregnant women receive at least one antenatal care (ANC) visit, and 44% receive at least four ANC visits and the full package of key interventions [1]. In addition to physical and health systems barriers, in many settings, women’s low status negatively impacts utilization of antenatal care services. As compared to men, women often lack decision-making power to allocate resources for health-care seeking [7, 8], particularly in contexts where men determine whether and under what conditions their spouses will use health services [9, 10]. This can prove problematic particularly in households where men underestimate the importance of antenatal care [1, 11]. Even following ANC consultation, women’s limited means and authority to implement healthy home practices have hindered development goals.

Global efforts to empower women have aimed to redress gender-based inequalities by implementing programs to increase opportunity, control, and inclusion for women [12]. Empowered women, particularly those who are more autonomous, have increased pregnancy health care-seeking [13, 14], are more likely to have skilled delivery attendance [13, 15], utilize modern contraceptive methods [16, 17], and have lower infant mortality [18]. At the same time, there has been increasing emphasis on encouraging greater male participation in women’s health [19, 20]. Inviting men to accompany women to ANC is considered an important strategy for reducing maternal morbidity and mortality by enabling them to sufficiently prepare for birth and avoid care-seeking delays for obstetric emergencies [21–23]. Men’s involvement in ANC is also intended to encourage husbands to support women’s care from pregnancy, to delivery, and throughout the postnatal period [1]. Research suggests that men’s presence during ANC can improve uptake of institutional deliveries [23], postnatal service utilization [24], and spousal communication [25]. However, socio-cultural norms that define pregnancy as a woman’s domain as well as health systems factors such as poor health worker communication and low male representation among staff have been shown to discourage men’s participation [11, 26].

Although in theory the two strategies can work synergistically, in practice they may result in opposing processes and outcomes. Women’s empowerment initiatives typically involve raising the status of women and shifting the gender balances of power [12, 27], while male involvement programs are meant to result in shared decision-making and the adoption of new male norms for constructive engagement [28]. However, in some cases, such programs have been criticized for inadvertently bypassing efforts to empower women and taking advantage of men’s superior status to achieve program goals [29]. Tensions exist, and there is little empirical evidence on the relationship between women’s empowerment and male involvement. Researchers describe a range of possible scenarios [30]. One constitutes the ideal situation where women’s autonomy and male involvement reinforce one another [30]. Less ideal situations occur when empowering women excludes male partners or involving men undermines women’s preferences [31].

No study to-date has examined links between women’s empowerment and male antenatal involvement in sub-Saharan Africa. In some communities, male partner attendance to ANC is rare, and empowering women may result in even fewer instances of engaging spouses [21, 32, 33]. In contrast, given that male social norms often discourage accompanying spouses to ANC [34], more empowered women may be better equipped to persuade spousal participation during pregnancy [3, 34, 35], or such behaviors may reflect emerging norms among couples exposed to recent health campaigns [9, 36, 37]. Understanding differences in male involvement by levels of women’s empowerment can help inform strategies aiming to address poor maternal health outcomes resulting from women’s unequal status and low spousal engagement.

### Women’s empowerment

The definition of empowerment has varied substantially in the literature, but is generally described as the “expansion in people’s ability to make strategic life choices in a context where this ability was previously denied to them” [38]. This conceptualization encompasses a process of change in which an individual acquires both resources and agency to make and act upon decisions that affect her well-being or that of others [27]. Terms such as women’s autonomy, power, status, and agency are embedded within the concept of empowerment, and are often used interchangeably in the literature [27, 39]. However, empowerment connotes more than independence of control from others. It additionally represents gaining greater choice and capacity to affect significant life outcomes [27, 40]. In this light, empowered women can more successfully negotiate their reproductive and health-related preferences with male partners [41].

Measuring empowerment has proven difficult for several reasons. One challenge is that empowerment is a latent construct that cannot be directly observed, and less is known regarding the intrinsic causal processes [42]. It is thus inferred by a set of observable indicators, such as decision-making, financial independence, or mobility freedom, which are considered representative, in part, of the effects of empowerment [43]. Empowerment is also multidimensional in that women can be empowered (or disempowered) in several life domains [39, 44]. Common dimensions include economic, socio-cultural, familial and interpersonal, legal, political, and psychological [27, 44, 45] and are represented across the literature as intersecting variants of choice, control, and power [27]. At the same time, however, empowerment dimensions may be conceptualized differently depending on the context, and even in similar settings, women may experience some dimensions and not others [43, 46].

Despite these challenges, the increased recognition on the importance women’s empowerment in global health and development has led to the development of a large body of research. Since the late 1990s, the Demographic and Health Survey (DHS), which collects nationally representative data among women of reproductive age (15 to 49 years) in lower- and middle-income countries, has incorporated indicators of women’s empowerment intended to have broad applicability. Such measures have provided substantive empirical evidence on the association between women’s empowerment and reproductive health outcomes in sub-Saharan Africa [35, 46–49].

### Study objectives

This study assesses whether a woman’s empowerment status, in sum and across dimensions, is associated with the odds that her male partner accompanied her to at least one facility-based antenatal consultation.

## Methods

### Country selection

Eight sub-Saharan African countries were included in the analysis: Burkina Faso, Burundi, Malawi, Mozambique, Rwanda, Senegal, Uganda, and Zimbabwe. Countries were selected based on having met each of the following inclusion criteria: having a sufficient sample size of married or cohabiting women, having recently conducted a DHS in the year 2010 or later, having included a men’s survey measuring antenatal accompaniment, and having similar women’s empowerment questions regarding household decision-making, control over earnings, attitudes towards domestic violence, and asset ownership.

### Country settings

The inclusion of these countries provides a diverse, yet comparable landscape for examining women’s empowerment in relation to male involvement. According to the United Nations Development Programme (UNDP), sub-Saharan Africa has the highest gender inequity in the world, as measured by aspects of reproductive health and women’s participation in government, higher education, and the labor market [50]. The UNDP gender inequality index represents the loss in human development due to gender inequality, where 0 represents full equality and 1 indicates the lowest possible status for women. Rwanda (.414) and Burundi (.476) have relatively less gender inequality compared to other countries, while Burkina Faso (.609) and Mozambique (.582) have the highest gender inequality indices [50]. According to the DHS 2010–2011, attendance to at least one ANC visit is nearly universal (>90%) in all eight countries [51]. However, Burundi and Burkina Faso have the poorest coverage rates of the recommended four ANC visits at 33.4% and 33.7% of pregnant women, respectively, with the highest rates in Mozambique (50.6%) and Zimbabwe (64.8%) [51]. Access to and utilization of maternal health services (which often requires the support of male spouses) are also relatively low across countries. Malawi has the highest percentage of women delivering with a skilled birth attendant (71.4%), while the lowest skilled birth attendance rates are in Mozambique (54.3%) and Uganda (50.6%) [51].

### Sample selection

To examine the association between women’s empowerment (measured in the women’s survey) and male accompaniment to ANC (measured in the men’s survey), we used the freely-available DHS matched couples’ dataset where the couple is the unit of analysis. The couples’ dataset represents completed interviews of a subset of men and women within a single household who declared each other as married or cohabiting partners. To determine the analytical sample, we included women aged 15 to 49 from the couples’ dataset who had given birth within two years prior to the survey, reported at least one antenatal visit during their most recent pregnancy, and who had non-missing data for all empowerment items and the outcome. Country-level analytic samples included: Burkina Faso (n = 2,490), Burundi (n = 1,042), Malawi (n = 1,353), Mozambique (n = 414), Rwanda (n = 1,211), Senegal (n = 505), Uganda (n = 428) and Zimbabwe (n = 459).

### Outcome measure

Male accompaniment to ANC was the primary outcome of the analysis. In the DHS, the respondent was asked if he was ever present during any of his partner’s antenatal check-ups for their youngest child. The response options were: present (code = 1) and not present (code = 0).

### Explanatory variable

Women’s empowerment was measured as a composite score, ranging from 0 to 9, where 0 represents “not empowered” and 9 represents “highly empowered”. Three dimensions were included based on available DHS items: economic, socio-familial, and legal. Each woman’s score was calculated from the sum of 9 individual questions scored 0 (=not empowered) or 1 (=empowered). Table 1 summarizes the aggregation rules used to code the more empowered response of each question. Using the framework proposed by Malhotra et al., [27], the empowerment dimensions and their respective items were conceptualized as follows: *Economic empowerment* referred to access to and control over economic resources and participation in economic markets. The dimension was examined using one question concerning women’s income relative to her partner (code = 1 if more, about the same, or woman is sole earner; code = 0 if less than partner or women/neither earn cash) and three questions relating to decisions on the woman’s income, partner’s income, or household purchases (code = 1 if woman decides alone or jointly; code = 0 if partner decides).

**Table 1:** **DHS empowerment items and aggregate codes**

Dimension	Item label	DHS question	DHS response categories	Aggregate recodes used in analysis ^a^
Economic	Women’s income relative to partner	Would you say that the money you earn is more than what your partner earns, less, or about the same?^b^	1 = more than him;	Code = 1 if Q1 = 1 or 3
			2 = less than him;	*Code = 1* if she’s sole earner
			3 = about the same;	Code = 0 if Q1 = 2^e^
			4 = partner doesn’t bring in money	*Code = 0* if woman does not earn, partner does
				*Code = 0* if neither earn
	Decision on woman’s income use	Who usually decides how the money you earn will be used?^b^	1 = respondent	Code = 1 if Q2 = 1 or 2
			2 = jointly	Code = 0 if Q2 = 3-6^c,e^
			4 = partner	*Code = 0* if does not earn cash
	Decision on partner’s income use	Who usually decides how your partner’s earnings will be used?	1 = respondent	Code = 1 if Q3 = 1 or 2
			2 = jointly	Code = 0 if Q3 = 3-7^c,e^
			4 = partner	
			7 = partner doesn’t bring in money	
	Decision on household purchases	Who usually makes decisions about major household purchases?	1 = respondent	Code = 1 if Q4 = 1 or 2
			2 = jointly	Code = 0 if Q4 = 3-6^c,e^
			4 = partner	
Socio-familial	Decision on family visits	Who usually makes decisions about visits to your family or relatives?	1 = respondent	Code = 1 if Q5 = 1 or 2
			2 = jointly	Code = 0 if Q5 = 3-6^c,e^
			4 = partner	
	Decision on own health care	Who usually makes decisions about health care for yourself?	1 = respondent	Code = 1 if Q6 = 1 or 2
			2 = jointly	Code = 0 if Q6 = 3-6^c,e^
			4 = partner	
	Attitudes on partner violence	Is a husband justified in hitting/beating his wife in the following situations?^d^	1 = yes	Code = 1 if all five Q8’s = 0
			0 = no	Code = 0 if at least 1 Q8 = 1
Legal	Home ownership	Do you own this or any other house?	0 = does not own	Code = 1 if Q9 = 1, 2, or 3
			1 = alone only	Code = 0 if Q9 = 0^e^
			2 = jointly only	
			3 = alone & jointly	
	Land ownership	Do you own any land? (DHS,	0 = does not own	Code = 1 if Q10 = 1, 2 or 3
			1 = alone only	Code = 0 if Q10 = 0^e^
			2 = jointly only	
			3 = alone & jointly	

^a^Due to skip pattern, italicized codes are investigator-derived from combinations of other DHS questions. ^b^Question is skipped if respondent woman does not earn cash. ^c^Codes not shown: 3 = respondent and other; 5 = someone else; 6 = other; 8 = don’t know. ^d^If she: goes out without telling him, neglects the children, argues with him, refuses sex, or burns food. [e] Includes 8 = don’t know.

*Socio-familial empowerment* is often characterized as women’s freedom of mobility and power balance within social networks. It additionally represents women’s familial and marital roles, including household status in contexts of conflict and negotiation. Three questions were included relating to decision-making for woman’s health care (code = 1 if woman decides alone or jointly; code = 0 if partner decides), decisions about visits to family or relatives (code = 1 if woman decides alone or jointly; code = 0 if partner decides), and attitudes on partner violence (code = 1 if beating wife is not justified for all five scenarios, otherwise code = 0). The five domestic scenarios included: goes out without telling him, neglects the children, argues with him, refuses to have sex, or burns food. *Legal empowerment* signified women’s judicial and legislative entitlements, including land and property rights. Two DHS questions measured this dimension when respondents were asked if they owned a house or any land. A code of 1 was assigned to women who had any sole or joint ownership of a house or land. Women who did not own property were coded as 0.

### Adjustment variables

Prior research has shown that education [46], employment and economic involvement [46, 52], wealth [53], and number of currently living children [31, 46] are associated with women’s empowerment. In addition, parity [33], number of surviving children [30], maternal age [30, 34], men’s occupation [30, 34, 36, 54], religion [36], education level [30, 34, 54], and wealth/standard of living [30, 54] have been shown to be associated with male involvement in pregnancy care. In our analysis, potentially confounding variables that were included in the multivariable analysis were: maternal characteristics (maternal age in years, highest level of education completed, number of living children, and religious affiliation); male partner characteristics (age in years, highest level of education completed); and household characteristics (residence and wealth quintile).

### Analysis

Data were analyzed using STATA Version 13.1 (Stata Corporation, College Station, TX). First, we described the sample characteristics for each country using DHS sample weights, adjusting for differences in the probability of selection from the survey design. We also described the weighted distribution of the outcome and each of the women’s empowerment items. Second, a composite score of women’s empowerment was calculated based on the sum of the nine empowerment items with a total possible score ranging from 0 to 9. Women’s empowerment was categorized into two groups: the proportion of women with total scores above the country-specific mean versus those with scores at or below the country-specific mean. These steps were repeated for each dimension. These binary variables, characterizing high and low empowerment, were then used in country-specific bivariate and multivariable logistic regressions models to examine the association of women’s empowerment and antenatal male attendance. To examine the independent and potentially different relationships of dimensions of women’s empowerment on male involvement, we also fitted a combined model with all three dimensions. All analyses were adjusted for the complex survey design to correct the variance estimations. Odds ratios were considered statistically significant at *p* < .05. In addition, in the report of findings, this study adhered to the STROBE guidelines for cross-sectional studies.

### Ethical considerations

The proposed analysis was exempt as described in the guidelines issued by the Johns Hopkins Institutional Review Board.

## Results

### Sample demographic characteristics

Table 2 presents the sample demographic characteristics by country. The mean age for all women and their male partners was 29.3 and 36.0 years, respectively. The mean number of living children for all women was 3.4 (±0.03) with Senegal having the highest average (4.1 ± 0.1) and Zimbabwe having the lowest average (2.6 ± 0.1). The proportion of women with at least a primary education was highest in Zimbabwe (98.8%) and Uganda (87.5%) and lowest in Senegal (29.7%) and Burkina Faso (14.0%). Education patterns were similar across countries among male partners, and men were typically more educated than women. In each country, women reported a range of religious affiliations. Variability in reported religion was highest in Mozambique (65.4% Christian, 25.6% Muslim, 1.0% other, 8.0% none) and Burkina Faso (28.3% Christian, 61.2% Muslim, 9.4% other, 1.1% none). Urban residence was low in all eight countries, ranging from 7.3% in Burundi to 34.9% in Senegal.

**Table 2:** **Demographic characteristics**

Country	Burkina Faso	Burundi	Malawi	Mozambique	Rwanda	Senegal	Uganda	Zimbabwe
Year	2010	2010	2010	2011	2010	2010	2011	2010
Sample size	2,490	1,042	1,353	414	1,211	505	428	459
**Maternal Characteristics**								
Age (mean ± SE)	29.5	30.0	27.6	28.4	30.0	31.6	28.9	28.2
	(±0.2)	(±0.3)	(±0.2)	(±0.4)	(±0.2)	± 0.4	± 0.4	± 0.3
Number of living children	3.5	3.6	3.2	3.3	3.1	4.1	4.0	2.6
(mean ± SE)	(±0.1)	(±0.1)	(±0.1)	(±0.1)	(±0.1)	± 0.1	± 0.1	± 0.1
Primary education or higher (%)	14.0	43.1	84.4	65.1	82.8	29.7	87.5	98.8
Religion (%)								
Christian	28.3	94.1	88.8	65.4	97.9	4.6	88.1	92.4
Muslim	61.2	2.8	10.3	25.6	0.7	94.1	8.6	0.2
Other	9.4	1.5	0.2	1.0	0.7	1.3	3.3	1.3
No religion^a^	1.1	1.6	0.6	8.0	0.7	-	-	6.1
**Male Partner Characteristics**								
Age (mean ± SE)	39.0	35.4	32.9	33.7	33.9	42.4	34.5	33.6
	(±0.3)	(±0.3)	(±0.2)	(±0.6)	(±0.2)	(±0.5)	(±0.5)	(±0.4)
Primary education or higher (%)	19.1	56.5	92.6	85.0	83.4	36.1	95.0	99.1
**Household Characteristics**								
Urban residence (%)	13.5	7.3	10.5	24.3	9.1	34.9	12.0	34.7
Wealth index (%)								
Poorest	18.9	19.3	16.7	26.8	19.8	20.8	20.5	18.0
Poorest	22.6	22.9	25.0	24.5	22.6	26.2	22.1	17.4
Middle	22.6	21.1	22.8	19.4	22.2	19.3	20.0	22.0
Richer	24.7	20.1	19.7	17.1	22.2	20.8	20.7	22.3
Richest	11.3	16.6	15.8	12.2	13.1	12.8	16.7	20.3

^a^‘No religion’ is reported in countries with this option.

### Sample empowerment characteristics

Table 3 shows the descriptive statistics for the nine women’s empowerment indicators by country. The weighted total empowerment score was highest in Zimbabwe (6.3 ± 0.1) and Rwanda (5.7 ± 0.01) and lowest in Senegal (2.8 ± 0.1) and Burkina Faso (2.6 ± 0.1). By dimension, Zimbabwe had the highest proportion of women responding affirmatively for all of the economic items. Thirty-five percent (35.4%) of women earned income equal to or greater than their male partner, and the majority of women (85-90%) reported having a say in decisions about their own income, the income of their male partner, or major household purchases. In contrast, Burkina Faso and Burundi had the two lowest economic empowerment responses. Approximately 2-4% of women reported earning income equal to or greater than their male partner, respectively. Burundi had the lowest proportion of women (11%) who reported having a say on use of their own income, while women in Burkina Faso had the lowest proportions of women (6 and 16%, respectively) having a say in decisions on use of their partner’s income or household purchases. This pattern was consistent among the socio-familial indicators: 85-86% of women in Zimbabwe vs.18-48% of women in Burkina Faso and Burundi reported having a say on decisions on family visits and health care, respectively. The proportion of women opposed to all domestic violence justifications was highest in Malawi (86.5%) and lowest in Burundi (26.5%). For the legal dimension, the proportion of women owning a house or land was lowest in Senegal (12.9% and 16.5%, respectively). Ownership of a house among women was highest in Burundi (88.2%) and Mozambique (88.1%), while the proportion of women jointly or solely owning land was highest in Rwanda (83.2%).

**Table 3:** **Distribution of women’s empowerment indicators and male accompaniment to ANC by country**

Women’s empowerment indicators	Burkina Faso	Burundi	Malawi	Mozambique	Rwanda	Senegal	Uganda	Zimbabwe
Year	2010	2010	2010	2011	2010	2010	2011	2010
Sample size	2,490	1,042	1,353	414	1,211	505	428	459
Percent (%) women responding affirmatively								
**Economic**								
Earns income ≥ male partner	2.0	4.1	13.6	8.1	21.2	11.1	13.0	35.4
Has say on own income use	37.9	11.4	25.7	21.9	57.2	73.2	55.2	87.4
Has say on partner’s income use	5.5	63.2	29.1	48.1	69.4	21.4	48.6	85.0
Has say on household purchases	15.7	53.1	28.1	58.2	66.8	30.3	57.0	90.4
*Mean economic sub-score*	0.6	1.3	1.0	1.4	2.1	1.4	1.7	3.0
(out of 4 total points) (±SD)	(±0.03)	(±0.04)	(±0.04)	(±0.1)	(±0.04)	(±0.1)	(±0.1)	(±0.1)
*%* women > weighted mean	45.3	48.1	53.5	45.6	44.0	37.7	57.4	79.6
**Socio-familial**								
Has say on visits to family	48.6	75.6	60.9	72.7	80.6	44.6	59.0	85.4
Has say on decisions about								
health care for herself	17.5	75.7	51.7	70.4	72.1	33.9	60.5	86.1
Agrees domestic violence by								
husband is never justified	52.9	26.5	86.5	70.6	40.9	33.0	43.1	55.0
*Mean socioc-familal sub-score*	1.2	1.8	2.0	2.1	1.9	1.1	1.6	2.3
(out of 3 total points) (±SD)	(±0.03)	(±0.03)	(±0.03)	(±0.1)	(±0.03)	(±0.1)	(±0.1)	(±0.1)
*%* women > weighted mean	32.9	68.7	66.8	40.9	73.2	36.8	58.2	45.2
**Legal**								
Owns house alone or jointly	39.0	88.2	82.5	88.1	83.4	12.9	64.5	52.3
Owns land alone or jointly	43.6	79.0	52.1	79.1	83.2	16.5	59.3	53.0
*Mean legal sub-score*	0.8	1.7	1.3	1.7	1.7	0.3	1.2	1.1
(out of 2 total points) (±SD)	(±0.03)	(±0.03)	(±0.03)	(±0.03)	(±0.02)	(±0.1)	(±0.1)	(±0.1)
*%* women > weighted mean	52.5	74.3	44.3	75.0	75.6	19.8	47.8	44.3
**Total Empowerment Score**	2.6	4.8	4.3	5.2	5.7	2.8	4.6	6.3
[9 total points]; mean (±SD)	(±0.1)	(±0.1)	(±0.1)	(±0.1)	(±0.1)	(±0.1)	(±0.1)	(±0.1)
% women > weighted mean	49.2	59.8	42.6	46.7	61.8	48.9	53.7	48.9
**Male partner ANC accompaniment (%)**	45.2	18.2	41.0	44.2	86.8	31.8	49.7	32.4

### Prevalence of male partner accompaniment to ANC

Slightly less than half (45.7%) of all men in the study population reported being present during one of his partner’s antenatal check-ups. There were considerable differences by country (Table 3). Rwanda had the highest proportion of men who accompanied their partners to ANC (86.8%) while Burundi had the lowest proportion (18.2%). In Senegal and Zimbabwe, roughly a third of men (32%) were present during at least one check-up, with slightly higher accompaniment rates in the remaining countries: Malawi (41.0%), Mozambique (44.2%), Burkina Faso (45.2%), and Uganda (49.7%), respectively.

### Women’s empowerment and partner accompaniment to ANC

Table 4 presents the odds ratios (OR) and 95% confidence intervals (CI) of male accompaniment to ANC by women’s composite and dimensional empowerment status. A range of scenarios were observed across countries. Women with higher empowerment (composite scores above the country mean level) had significantly higher odds of seeking ANC with their male partner as compared to women with lower empowerment (composite scores below the country mean level) in Burkina Faso (OR = 1.27, 95% CI: 1.08, 1.50) and Uganda (OR = 1.53, 95% CI: 1.00-2.35). A significant positive association was also observed in the economic domain in Burkina Faso (OR = 1.24, 95% CI: 1.08-1.47). Although similar to Uganda, no significant relationship was observed in Burkina Faso among remaining empowerment dimensions. In contrast, in Malawi, women’s higher empowerment status was significantly associated with lower odds of male accompaniment to ANC both among women with above average composite empowerment scores (OR = 0.77, 95% CI: 0.62-0.97) and above average economic empowerment scores (OR = 0.75, 95% CI: 0.59-0.94). The negative trends in more empowered women being less likely to be accompanied to ANC by spouses persisted in the socio-familial (OR = 0.89, 95% CI: 0.69-1.14) and legal domain (OR = 0.94, 95% CI: 0.75-1.17), although the association was not statistically significant. No significant differences were observed in the odds of male antenatal accompaniment by women’s composite empowerment status in Burundi (OR = 0.94, 95% CI: 0.67, 1.31), Mozambique (OR = 0.94, 95% CI: 0.61, 1.45), Rwanda (OR = 1.35, 95% CI: 0.95, 1.91), Senegal (OR = 1.13, 95% CI: 0.77, 1.66), or Zimbabwe (OR = 1.02, 95% CI: 0.66, 1.57). These countries also had similarly null findings across the dimensional empowerment components.

**Table 4:** **Odds ratios and 95% confidence intervals of male accompaniment to ANC by women’s empowerment status**

OR (95% CI) by empowerment status ^A^	Burkina Faso	Burundi	Malawi	Mozambique	Rwanda	Senegal	Uganda	Zimbabwe
Year	2010	2010	2010	2011	2010	2010	2011	2010
Sample size	2,490	1,042	1,353	414	1,211	505	428	459
**Women’s Empowerment** *(Unadjusted Model)* ^B^								
Composite	1.32*	1.00	0.75*	0.83	1.3	1.11	1.78*	0.94
	(1.13, 1.55)	(.72, 1.37)	(.60, .94)	(.56, 1.22)	(.93, 1.82)	(.77, 1.60)	(1.21, 2.61)	(.64, 1.39)
Economic	1.30*	1.20	0.72*	0.91	1.24	1.27	1.40	1.39
	(1.10, 1.53)	(.85, 1.71)	(.57, .91)	(.58, 1.42)	(.87, 1.78)	(.83, 1.96)	(.91, 2.15)	(.82, 2.35)
Socio-familial	1.03	1.22	0.86	0.83	1.11	0.82	1.37	1.27
	(.86, 1.23)	(.82, 1.80)	(.68, 1.11)	(.53, 1.30)	(.75, 1.63)	(.53, 1.28)	(.89, 2.11)	(.85, 1.89)
Legal	0.91	0.81	0.97	1.70*	1.30	1.01	1.08	0.79
	(.78, 1.07)	(.58, 1.14)	(.77, 1.21)	(1.09, 2.66)	(.90, 1.88)	(.65, 1.58)	(.73, 1.61)	(.53, 1.18)
**Women’s Empowerment** *(Adjusted Model)* ^B,C^								
Composite	1.27*	0.94	0.77*	0.94	1.35	1.13	1.53*	1.02
	(1.08, 1.50)	(.67, 1.31)	(.62, .97)	(.61, 1.45)	(.95, 1.91)	(.77, 1.66)	(1.00, 2.35)	(.66, 1.57)
Economic	1.24*	1.12	0.75*	1.22	1.24	1.22	1.33	1.33
	(1.08, 1.47)	(.78, 1.61)	(.59, .94)	(.73, 2.05)	(.86, 1.78)	(.78, 1.92)	(.85, 2.09)	(.78, 2.27)
Socio-familial	1.00	1.13	0.89	0.91	1.18	0.87	1.30	1.02
	(.83, 1.20)	(.75, 1.69)	(.69, 1.14)	(.57, 1.46)	(.80, 1.76)	(.55, 1.37)	(.82, 2.06)	(.66, 1.59)
Legal	0.94	1.16	0.94	1.32	1.38	1.01	0.97	1.02
	(.79, 1.10)	(.79, 1.71)	(.75, 1.17)	(0.80, 2.18)	(.93, 2.04)	(.63, 1.60)	(.63, 1.50)	(.61, 1.71)

^A^Reference group (OR = 1) includes women with scores below the country mean level; ^B^Scores only adjusted for cluster survey design; ^C^Scores additionally adjusted for maternal, male partner, and household characteristics. *Significant at p < .05.

## Discussion

To our knowledge, this study is the first to-date to examine the relationship between women’s empowerment and male antenatal accompaniment in sub-Saharan Africa. We build upon prior analyses by including a composite empowerment measure in addition to examining the direct contribution of three dimensions (economic, socio-familial, and legal), using analytical methods that account for differences in individual, partner and household characteristics. Most studies examining women’s empowerment have focused on a single country or up to four countries. However, this analysis provides a more geographically diverse sample of eight countries using country-specific estimates to inform our understanding across the region. Key findings indicated that male ANC accompaniment was not uncommon. However, we observed mixed results regarding the association between women’s empowerment and male accompaniment to ANC. In some settings, women’s empowerment was positively and significantly associated with increased odds of male attendance, predominately in the economic and legal domain. However, this relationship was not universal. In several cases, no associations were observed or negatively associated in which more empowered women were significantly less likely to have their spouse’s presence at ANC. The analysis’ mixed results of positive, negative, and null associations reveal several important programmatic and research implications.

First, the significant positive association of women’s empowerment with male involvement is encouraging as it suggests increasing women’s participation in economic and legal domains works synergistically with male involvement strategies meant to enhance shared decision-making. Positive associations were found in overall empowerment in Uganda and Burkina Faso, and in the economic empowerment dimension in Burkina Faso. In these countries, women who had a say in more decisions on use of household resources were more likely to be accompanied to ANC by their spouses as compared to women who were less often included in these decisions. This pattern is similar to those observed by Mullany et al., [30] in Nepal where joint-decision making among women was significantly associated with greater male participation during pregnancy. Reasons for this positive association in our study may be that more empowered women were more likely to negotiate and involve their male partners in prenatal care-seeking. It is also possible that couples accustomed to shared decision-making in other life domains were more likely to view pregnancy care as a shared domain. In addition, women who were sole decision-makers may also be more capable of soliciting spousal antenatal assistance than women excluded from participating in other aspects of family life. Conversely, women with limited say or participation may select not to invite spouses who may otherwise restrict their care-seeking choices. In a recent review, Ditekemena et al., [34] found that some African women feared violence from spouses who accompanied them to ANC, particularly as a result of antenatal HIV testing. The significant positive observations imply that male involvement efforts may benefit from both empowerment initiatives that promote women’s participation in social and economic spheres and male-centered approaches that focus on new male norms. In addition, increased male involvement in ANC may spur changes in social norms for women and men. Such efforts may be most impactful in settings where women’s empowerment scores are lowest, as was the case in Burkina Faso.

A second implication relates to the significant negative relationship between empowerment and antenatal accompaniment in Malawi. The negative trends in Malawi were observed among all empowerment dimensions, although the relationship was only significant for the combined and economic empowerment scores. Thapa & Niehof [31] also found in Nepal that increased women’s autonomy was associated with lower likelihoods of husband’s presence at ANC. In our analysis, one explanation for the reverse relationship could be that women with greater participation in health care and household decisions, including asset ownership, saw less of a need to invite spouses to ANC. It may be also that such an invitation was viewed as unnecessarily overlapping with women’s roles. Kululanga et al., [55] found in Malawi that some women viewed male involvement in pregnancy as a “foreign concept” and synonymous with an infringement on “territory they did not want men to invade”. Thus, women with more influence in household matters may also be more likely to voice and achieve their preferences. This suggests that enabling women and their partners to identify potentially beneficial and acceptable norms of male participation during pregnancy may assist in implementing approaches that do not undermine women’s autonomy. Alternatively, higher empowerment scores could indicate male partner absence, such as a spouse who works abroad, where women alone bear the burden of decision-making out of necessity – which is not the aim of empowerment approaches. Kululanga et al., [55] also found in Malawi that male involvement programs were sometimes perceived as unfair to unmarried or otherwise single women and perpetuated cultural norms that men were superior. In this regard, male involvement strategies may more appropriately empower women by identifying other peers or relatives who can assist in preparing for birth and support women’s continuity of care throughout the postpartum period.

A final implication relates to the lack of significant findings in Burundi, Mozambique, Rwanda, Senegal, and Zimbabwe. In Rwanda, an overwhelming majority of men reported accompanying their wives to ANC – the highest male attendance rate compared to other countries, while Burundi had the lowest accompaniment rate. Our findings suggest that the pervasive male norms in these settings are not substantially impacted by women’s empowerment status. Another possible explanation for the null findings may be that Rwanda and Zimbabwe also had the highest total women’s empowerment scores compared to other selected countries. For example, while empowerment scores were highest in Zimbabwe (6.3 out of 9), only a third of men reported attending at least one antenatal check-up with their spouse. Zimbabwe also had the highest proportion of women receiving all four recommended ANC visits as compared to other selected countries, which could indicate a stronger cultural norm that defines pregnancy as a woman’s domain. Male involvement in these countries may reflect other social and health systems factors not captured in the DHS empowerment measure.

Additional qualitative research may provide more in-depth understanding of the contexts driving the different associations between women’s empowerment and male antenatal accompaniment. Qualitative methods could also assist in examining whether there is indeed no association between women’s empowerment and joint antenatal care-seeking, or whether women’s empowerment aspects which are linked to male involvement were unmeasured in this analysis. Measuring a latent construct such as empowerment across a range of contexts is inherently challenging, and it is possible that different meanings and manifestations of empowerment were unable to be captured by the DHS. Some researchers have increasingly questioned the adequacy of DHS empowerment measures for use in sub-Saharan Africa, particularly since the items were developed based on experiences and conceptual models drawn from Asia [35, 49]. It is worth noting that in our analysis none of the socio-familial empowerment items were associated with male ANC accompaniment. Do & Kurimoto [35] also found that DHS sociocultural empowerment measures did not predict contraceptive use in the study’s four African countries [35]. This suggests that such indicators, as measured, may not signify empowerment or are not related to male partner engagement in African settings. Rather, supplemental qualitative research could inform the development of more culturally relevant and sensitive measures of women’s empowerment and its dimensions in African settings that often have less restrictive gender norms. Future research might also examine the extent to which male antenatal accompaniment mediates or modifies the relationship between women’s empowerment status and improved reproductive health outcomes.

### Limitations and strengths

The study’s limitations should be considered. Women whose male partners were not surveyed were excluded from the analysis given that the outcome could only be determined from the DHS men’s survey. The couples’ survey design was also restricted to married and cohabiting women, and male prenatal involvement was not ascertained among women who did not attend ANC. Therefore, the study’s findings may not be generalizable to all women of reproductive age in the selected countries. Our secondary analyses suggested that empowerment measures were similar among women who attended ANC compared to those who did not, with the exception of Zimbabwe and Burkina Faso where women with higher empowerment scores were less likely to attend ANC. Further qualitative research would help to elucidate the relationship between male involvement in pregnancy and women’s empowerment among this cohort. It is also possible that ANC accompaniment measures may have varied if asked of women themselves rather than their male partners.

The study was also limited by the cross-sectional nature of DHS data. The cross-sectional measures did not capture the dynamism of empowerment or women’s or couple’s prior or accumulated experiences over time. In addition, causal associations between women’s empowerment and male accompaniment to ANC cannot be inferred. It is equally conceivable that women’s interpretation and response to the empowerment questions varied across settings, and some aspects of women’s empowerment may not have been captured by the indicators assessed in the DHS. Ultimately, this may have decreased the predictive value of the construct. Finally, use of a summative index has been challenged by some researchers for discounting item-level distinctions. We considered the summative index a more appropriate approach in examining the broader role of women’s empowerment, particularly given the concerns regarding the adequacy of any single item in an African context. Despite these limitations, the study’s strengths are the use of multiple empowerment indicators and dimensions, use of geographically diverse and representative samples, and inclusion of measures compatible with the existing literature.

## Conclusion

This study is the first to-date to examine the relationship between women’s empowerment and male antenatal accompaniment in sub-Saharan Africa. Our findings contribute to the existing literature by providing positive and negative empirical evidence on the relationship of potentially synergistic and opposing women’s empowerment and male antenatal accompaniment. However, results were mixed. Several null associations suggest that more qualitative and longitudinal research is needed to inform the development of locally relevant measures of women’s empowerment and its dimensions in African settings.

## Abbreviations

ANC: Antenatal care
CI: Confidence interval
DHS: Demographic health survey
OR: Odds ratio.
